# Evaluating the Efficiency of Municipal Solid Waste Management in China

**DOI:** 10.3390/ijerph15112448

**Published:** 2018-11-02

**Authors:** Qing Yang, Lingmei Fu, Xingxing Liu, Mengying Cheng

**Affiliations:** School of Management, Wuhan University of Technology, Wuhan 430070, China; yangq@whut.edu.cn (Q.Y.); mlsdb@whut.edu.cn (L.F.); chengmengying@whut.edu.cn (M.C.)

**Keywords:** municipal solid waste, three-stage data envelopment analysis, management efficiency, pilot cities, sustainable construction

## Abstract

Poor public health is always associated with the mismanagement of municipal solid waste (MSW). Many cities are besieged by MSW in the world. It is essential to do a good job in MSW management (MSWM). In order to improve the efficiency of MSWM, the Chinese government has intensively implemented relevant policies. There are still few studies on MSWM efficiency in China. The research aims to comprehensively analyze MSWM efficiency, find high-efficiency MSWM policy implementation routes and the breakthrough on improving MSWM efficiency. To measure Chinese MSWM efficiency accurately, this paper introduced the three-stage data envelopment analysis (DEA) model into the research. According to the results of DEA, Fuzzy c-Means algorithm was used to the cluster analysis of 33 typical cities. After eliminating the interference of the external environment and random disturbance, the mean value of MSWM efficiency declined from 0.575 to 0.544. The mean of pure technical efficiency (PTE) was declined from 0.966 to 0.611, while the mean of scale efficiency (SE) increased from 0.600 to 0.907. The PTE of central and northeastern cities was relatively low. The SE of western cities was comparatively high and the efficiency distribution of the eastern region was relatively scattered. In general, MSWM efficiency is low and expected to be improved. Regional differences in MSWM efficiency have been shown. The management effectiveness of eight pilot cities (MSW classification) is affirmative but not that significant. To improve MSWM efficiency, differential management for four types of cities should be carried out.

## 1. Introduction

Many cities are besieged by municipal solid waste (MSW) in the world, particularly in the cities of developing countries [1]. MSW is defined as local waste generated by households and commercial and governmental enterprises. It includes packaging, food waste, grass clippings, clothing, paper and other solid forms of waste, but does not include hazardous and infectious waste or sewage [2]. MSW management (MSWM) is known to be an important contributor to various environmental and public health problems [3]. It involves activities associated with generation, storage, collection, transfer and transport, processing and disposal of MSW [4]. But, in most cities, the MSWM system is comprised only of four activities: delivery, collection, transportation, disposal [5]. The objectives of MSWM are considering human safety, resource conservation and the reduction of, as much as possible, the environmental burdens of MSWM (energy consumption, pollution of air, land and water and loss of amenity) [6]. MSWM is a challenge and expensive task for the cities’ authorities worldwide [7]. Municipalities of developing countries usually spend 20 to 50% of their available municipal budget on MSWM, which affords only less than 50% of the population [8]. 

The formidable rise in MSW generation requires suitable management systems [9]. The mismanagement of MSW is not conducive to improving eco-efficiency [10]. Therefore, many countries attach more importance to MSWM [11]. MSWM efficiency has been one of the indicators to measure the level of urban governance [12]. It can reflect the degree of urban ecological civilization [13]. Eco-efficiency is typically defined as the ratio of added economic value to environmental impact or the ratio of added environmental benefits to the economic costs [14]. And the true MSWM efficiency is the efficiency of eliminating the interference of external environment and random disturbance. Nowadays, the level of MSWM varies from city to city. The accurate evaluation of MSWM efficiency is significant and valuable for the policy makers [4]. 

MSWM is one of the major problems that affect China’s environmental quality and the sustainable development of cities. Severe environmental issues have aroused due to improper and inefficient disposal of MSW [15,16]. In 2004, China surpassed the United States as the world largest waste generator and it was predicted that in 2030, China will likely produce twice as much MSW as the United States [17]. In 2016, the quantity of MSW generation in 214 large and medium-sized cities of China was 188.51 million tons in 2016, and the quantity of MSW disposal in the 214 cities was 186.84 million tons [18]. According to *China Urban Construction Statistical Yearbook*, China’s per capital MSW collection and transportation was 1.19 kg/day in 2016 [19]. According to a report issued jointly by McKinsey and Company and the ocean conservation group Oceana, the average MSW collection rate in China was 65%. In rural areas, there were only 5%. At present, two-thirds of cities in China are confronted with the predicament of being surrounded by MSW. China has become one of the countries in the world which is seriously troubled by MSW [20].

Recently, Chinese government has intensively issued relevant policies to strengthen MSWM. In June 2000, the Ministry of Housing and Urban-Rural Development of People’s Republic of China (MOHURD) published *Notice on the Publication of Pilot Cities for Classified Collection of Municipal Solid Waste* [21]. Eight cities were identified as pilot cities for the classified collection of MSW, which officially kicked off the prelude of Chinese waste sorting pilot work. Subsequently, national-level policies were constantly released. *Classification and Evaluation Standard of Municipal Solid Waste* [22], *Measures for the Management of Municipal Solid Waste* [23], *Environmental Protection Tax Law of the People’s Republic of China* [24], *Notice on Promoting Classified Management of Municipal Solid Waste in Schools* [25], *Opinions on Innovation and Improvement of Price Mechanism for Promoting Green Development* [26] and so on.In China, more importance has been attached to MSWM. However, there are still some problems in every link of MSWM. For example, low participation rate [27], mixed collection and transportation [28], lack of technology in MSW disposal [29]. It is a base to accurately measure the efficiency of MSWM and find high-efficiency MSWM policy implementation routes for improving the whole process management level of MSW. Effective management of MSWM is an important task in realizing “beautiful China” [30].

Measuring MSWM efficiency has gradually become a research hotspot. Bosch et al. (2000) used various methods to analyze the technical efficiency of the waste collection services in 75 municipalities located in Spanish, such as DEA (data envelopment analysis), FDH (free disposal hull, a non-parametric approach). *Number of Containers, Total Number of Vehicles, Total Number of Direct Workers* were inputs. *Tons of Refuse of Organic Material Collected* was output [31]. Benito et al. (2010) adopted one stage DEA to evaluate the efficiency in the municipal sector of the Region of Murcia. As for waste collection, *Costs of Personnel, Current Transfers* were input indicators. *Number of Tons of Domestic Refusal Collected, Number of Tons of Industrial or Commercial Refusal collected, Number of Industries, Commercial Establishments and Houses in which Refuse is Collected Daily* were output indicators. Compared with police, culture, sports, green areas and water supply, the efficiency of waste collection was at an intermediate level [32]. Zhou et al. (2012) adopted one stage DEA model in 34 cities. They selected *Capital investment for MSW maintenance and construction, Fixed Assets Investment in the Public Facilities of Municipal Environmental Sanitation* as inputs. *Quantity of MSW Transported by Airtight Vehicle* and *MSW Harmless Treatment Rate* were outputs. The results showed that MSWM efficiency should be improved [33]. Cristóbal et al. (2016) adopted a three-stage DEA model to measure the efficiency of different processing methods of kitchen waste. They achieved some better combinations of disposal methods [34]. Ferronato et al. (2016) analyzed the situation of MSWM in La Paz, which was based on the Wasteaware benchmark indicators and waste flow analysis. The research selected *Waste Collection Coverage, Waste Captured by the MSWM and recycling systems* as quantitative indicators. The research revealed that the MSWM was not efficient with regard to collection, recycling, financial sustainability [10]. In addition, many researchers have analyzed the efficiency of MSWM by qualitative methods [8,35,36,37,38,39].

The review above shows that the researchers have done some research on the efficiency of MSWM previously. However, further research is still necessary. DEA has been rarely used to evaluate the whole process management efficiency of MSW [5,10,31,33,35]. And the existing research mainly adopted traditional DEA models [5,31,32,33]. The influence of environmental factors and random errors to MSWM efficiency has not been eliminated, so the accuracy of the calculated efficiency needs to be improved. Three-stage DEA is a method of eliminating external environment and random interference. Zhao et al. (2018) used three-stage DEA to measure total-factor energy efficiency in Belt and Road Initiative Countries. The indicator system including 4 input indicators, 1 output indicator and 3 environmental indicators was established. Three-stage DEA consists of three-stage analysis: starting with a traditional BCC model (the DEA model is proposed by Banker, Charnes and Cooper), continuing with a Stochastic Frontier Analysis (SFA) to exclude external environment and random interference, and concluding with traditional BCC-DEA using adjusted data from Stage 2 to estimate the real efficiency [40].

The research aims to comprehensively analyze the management effectiveness of MSWM, find high-efficiency MSWM policy implementation routes and the breakthrough on improving MSWM efficiency. The paper introduces the three-stage DEA model into the research. On the basis of previous research, this paper establishes a reasonable indicator system for evaluating MSWM efficiency. Taking 33 cities in China as samples, the paper measures the real efficiency after eliminating external environment and random interference in 2016. The influence of external environment on the efficiency of MSWM is analyzed. And then, the status of MSWM in an all-round way is carried out. The analyses of the management effectiveness of the pilot cities (MSW classification) and regional differences are included. Furthermore, we find the crux of low efficiency and put forward the effective countermeasures to improve MSWM efficiency.

## 2. Materials and Methods

### 2.1. Research Methods of the Three-Stage DEA Model

SFA of parametric method [41] and DEA of non-parametric method are two popular methods which are designed for the measurement of relative efficiency [42]. DEA is a statistical evaluation method to evaluate the relative efficiency of decision-making units (DMUs). It abandons the traditional subjective weighting and makes the results of efficiency evaluation more accurate and acceptable [43]. In recent years, China has attached much importance to sustainable construction. The MSWM in China must pursue environmental benefits as well as economic and social benefits, which determines that the indicator system for measuring MSWM efficiency in China is multi-input and multi-output. Because SFA is limited to the efficiency evaluation of a single output, DEA is especially advantageous when analyzing MSWM [15]. Therefore, DEA is used to evaluate the efficiency of MSWM in the paper [41].

With the deepening of scholars’ research on the DEA model, the methods of one-stage DEA, two-stage DEA, three-stage DEA and so on have been appeared [44,45,46]. One-stage DEA cannot eliminate external environment and random interference. Two-stage DEA is only a regression analysis of the influencing factors, which cannot adjust raw data and accurately calculate the efficiency value. Three-stage DEA is a method which combines traditional DEA with SFA. What is more, because it can eliminate the influence of environmental factors and random errors, the measurement of efficiency is more objective and accurate than one-stage DEA and two-stage DEA [47,48]. It is widely used to measure efficiency in many fields, such as environmental efficiency [49], agricultural water use efficiency [50], scenic spots operational efficiency [51], learning–teaching technical efficiency [52], industrial eco-efficiency [53], traffic police efficiency [54]. It can be seen that three-stage DEA method has become an important branch of DEA model system [40].

In 2002, three-stage DEA was first presented by Fried et al. It removes the influence of external environmental factors and random errors based on the traditional DEA method. Therefore, the measured efficiency value is more realistic and reliable [53]. The basic research thinking of three-stage DEA model is as follows:

Stage 1: Using traditional DEA model to measure the efficiency value of decision-making unit (DMU).

The controlling of input is easier than controlling the output. Hence, the paper adopts traditional input-oriented BCC model to analyze the initial input and output data of 33 cities. BBC model is described as:

(1) $$\begin{array}{c} \mathrm{Min}(\theta )\\ s.t.\{\begin{array}{l} \overset{K}{\underset{i=1}{\sum }}X_{i}\lambda_{i}\leq {\theta X}_{i0},\;\;i\;=\;1,\;2,\;\ldots .,\;K\\ \overset{K}{\underset{i=1}{\sum }}Y_{i}\lambda_{i}\geq W\;\;\\ \overset{K}{\underset{i=1}{\sum }}\lambda_{i}=1\\ \lambda_{i}>0 \end{array} \end{array}$$

where *X* denotes the input indicator variable matrix of each city, and *Y* represents the corresponding output indicator variable matrix. *K* is the number of DMU. *i* represents the *i*th DMU weight of the input variable *i*, whereas *W* is the efficiency [55]. The efficiency value calculated is the Technical Efficiency (TE), which can be decomposed into Pure Technical Efficiency (PTE) and Scale Efficiency (SE). That is, TE = PTE *×* SE. TE is a comprehensive measure and evaluation of the resource allocation ability and the resource efficiency in the case of variable returns to scale (VRS) [56]. PTE reflects the production efficiency of input factors at the optimal scale of DMU. It indicates the efficiency of input factors in use [17]. SE is affected by scale factors. It reflects the gap between actual scale and optimal production scale [57].

Stage 2: Using SFA regression model to adjust the Inputs.

The slacks of Stage 1 are composed of three parts: the influence of external factors, managerial inefficiencies, and random errors [49]. The SFA regression of Stage 2 aims to capture the influence of external factors and random errors on the efficiencies [58]. The efficiency value of MSWM analyzed in the first stage cannot truly reflect the current status of MSWM. To improve the accuracy of DEA measurement, it is necessary to use SFA model to eliminate the interference. Assuming that there are *I* inputs and *p* observable external environment variables, the multiple linear regression model is constructed as follows:

(2) $$\;S_{ik}=\;f\left(Z_{k};\;\beta_{i}\right)+v_{ik}+\mu_{ik};\;i=1,\;2,\;\ldots ,\;I;\;k\;=\;1,\;2,\ldots ,\;K\;$$

where $S_{ik}$ is the slack variable of the *i*th item of the *k*th DMU, $Z_{k}$ denotes the environmental variable, $\beta_{i}$ is the coefficient of environmental variable, and $f\left(Z_{k};\;\beta_{i}\right)$ represents the influence of environmental variable on $S_{ik}$. $v_{ik}+\mu_{ik}$ denotes the mixed error. Where $v_{ik}$ is the random disturbance. $\mu_{ik}$ represents the management inefficiency, which aligns N^+^(0, $\delta_{\mu i}^{2}$) distribution. $v_{ik}$ has no relation with $\mu_{ik}$. The paper defines ${\gamma \;=\;\delta }_{\mu i}^{2}{/(\delta }_{\mu i}^{2}{+\delta }_{vi}^{2})$, which is the proportion of the management inefficiency variance to the total variance. When *γ* tends to 1, it means that the influence of management factors is dominant. When *γ* tends to 0, it means that the influence of random errors is dominant [40].

Eliminating the effects of environmental variables and random errors from the mixed errors is important. To analyze the corresponding input of each city under the same objective conditions, the adjustment equation is as follows:

(3) $$\;X_{ik}^{*}{\;=\;X}_{ik}+\left\{max\left[f\left(Z_{k};\;\hat{\beta_{i}}\right)\right]-f\left(Z_{k};\;\hat{\beta_{i}}\right)\right\}\;+\;\left[max\left(\hat{v_{ik}}\right)-\hat{v_{ik}}\right]\;$$

where, $X_{ik}$ and $X_{ik}^{*}$ are respectively the input data before and after the adjustment of the *k*th city. $\left\{max\left[f\left(Z_{k};\;\hat{\beta_{i}}\right)\right]-f\left(Z_{k};\;\hat{\beta_{i}}\right)\right\}$ is the adjustment of the *i*th input of the city *k*, which make the *k*th city in the most unfavorable environment. $\left[max\left(\hat{v_{ik}}\right)-\hat{v_{ik}}\right]$ is the adjustment of the *i*th input of the city *k*th, which is used to adjust all DMUs to the same condition of nature, namely the most unfortunate condition of the samples. In the end, the cities are placed under the same objective conditions [40]. When the environmental factors and random errors are the same, managerial inefficiencies constitute the last factor.

Stage 3: Adjusted DEA model. The adjusted input data and raw output data are measured by traditional BCC model.

The models in Stage 1 and Stage 3 are based on the two separate samples. Since the environmental factors and random errors have been eliminated in Stage 2, the results of efficiency calculated in the third stage are a pure managerial factor that bears a more realistic reflection of managerial efficiency [58]. Applying the BCC model, an efficiency score (ranged 0–1) is assigned to every DMU. DMUs with the efficiency of 1 are the benchmark of DMUs with the efficiency score of less than 1 [58,59].

**Figure 1:**
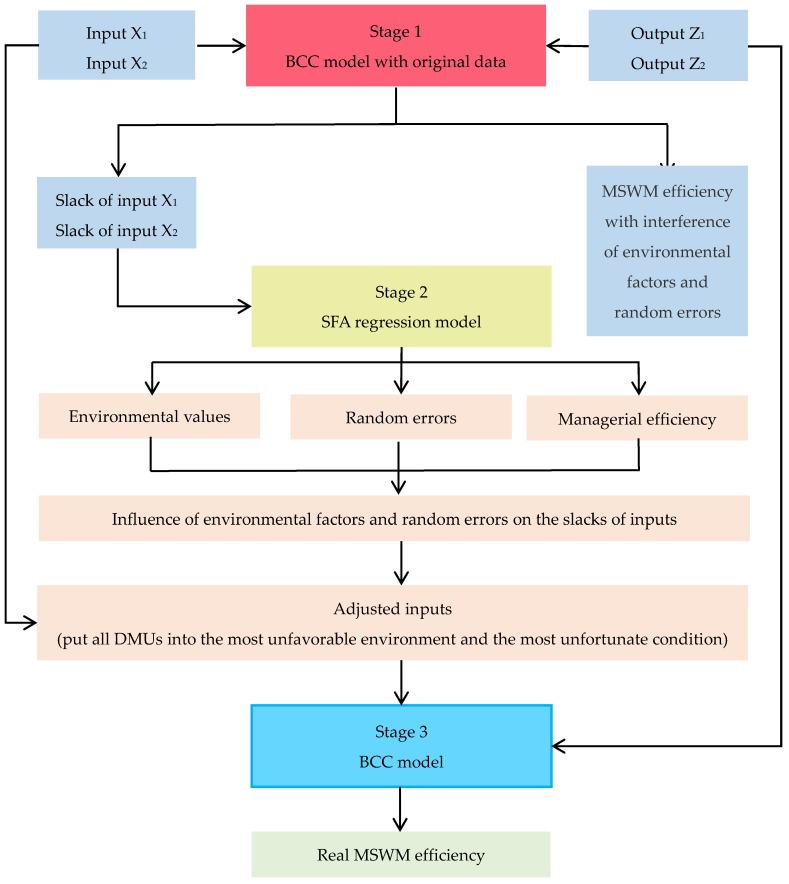
The framework of the established three-stage data envelopment analysis (DEA) model.

The framework of the established three-stage DEA model can be seen from Figure 1.

### 2.2. Research Methods of the Fuzzy c-Means Algorithm

In order to comprehensively identify the status in China, we used Fuzzy c-Means (FCM) algorithm to analyze whether there was regional difference in the efficiency of MSWM. FCM algorithm is a partition-based clustering algorithm, which can promote the maximum similarity between objects divided into the same cluster, and the smallest similarity between different clusters. In FCM, the degree of membership is used to determine the extent of each data point belongs to a certain cluster. It overcomes the defect of common C-means algorithm in which the data points are hard-divided.

FCM algorithm divides N data points into C clusters. To cluster data, it minimizes the objective function by iteratively updating the membership and cluster centers. The objective function is as follows:

(4) $$\begin{array}{l} \;J_{m}\left(U^{*}{,V}^{*}\right)\;=\;min\left[J_{m}\left(U,\;V\right)\right]\\ \;=\;min\left[\overset{n}{\underset{k=1}{\sum }}\overset{c}{\underset{i=1}{\sum }}{\left(u_{ik}\right)}^{m^{\prime }}{\left(d_{ik}\right)}^{2}\right]\\ \;s.t.\;\underset{i\in I_{k}}{\sum }u_{ik}=1\; \end{array}$$

where, U is m×n dimensional fuzzy partition matrix, V is  c×m dimensional cluster center matrix. $u_{ik}$ is the membership value of the *k*th data point in the *i*th cluster, m is the number of features. $m^{\prime }$ is weighting parameter varying in the range [1,∞], and *d* is the Euclidean distance between the data point and the data center. $U^{*}$ is partition matrix with optimal values, v* is cluster center matrix involving optimal cluster centers [60]. Cluster centers are calculated using following formulation:

(5) $$\;v_{ij}=\frac{\sum_{k=1}^{n}u_{ik}^{m^{\prime }}x_{kj}}{\sum_{k=1}^{n}u_{ik}^{m^{\prime }}}\;i=1,2,\ldots ,c;\;j=1,2,\ldots ,m\;$$

where, x is fuzzy variable describing data point. In essence, fuzzy partitioning is performed through an iterative optimization utilizing following formulation:

(6) $$\;u_{ik}\left(s+1\right)=\frac{1}{\sum_{j=1}^{c}{\left[\frac{d_{ik}(s)}{d_{jk}(s)}\right]}^{\frac{2}{m^{\prime -1}}}}\;$$

Formulas (5) and (6) are iterated until the optimal membership and cluster center are obtained [60].

### 2.3. Indicator Selection

Scientific selection of input and output indicators is the key to an efficiency measurement. Based on the scientific-nature of indicators and the feasibility principle of data indicator, this paper not only drew on previous research, but also considered the characteristics of MSWM to select relevant indicators. The selected indicators are shown in Table 1.

#### 2.3.1. Selection of Input Indicators

According to the comprehensive, scientific and representative principles in indicator selection, input indicators for measuring the efficiency of MSWM should cover the manpower, physical and financial resources in the four links of delivery, collection, transportation and disposal. Manpower resources can be measured by the number of relevant practitioners in MSWM, such as the number of sanitation workers, MSW sorters, the number of refuse collection and transportation personnel [31]. Physical resources can be reflected by the number of transporters for MSW [31,33]. Financial resources can be measured by the fixed assets investment in public facilities of MSW disposal.

At present, there are no authoritative data on the personnel related to MSWM in many cities. We cannot get the manpower input [33]. Based on the evaluation indicator system constructed by previous scholars, *Number of Vehicles and Equipment Designated for Municipal Environmental Sanitation* was taken as the physical resources invested in each city [33]. Due to the lack of data on fixed assets investment in the construction of public facilities for MSW disposal in some cities. *Fixed Assets Investment in the Public Facilities of Municipal Environmental Sanitation* was used to measure the financial input. Therefore, considering the feasibility principle, *Number of Vehicles and Equipment Designated for Municipal Environmental Sanitation* and *Fixed Assets Investment in the Public Facilities of Municipal Environmental Sanitation* were selected as input indicators to measure MSWM efficiency.

#### 2.3.2. Selection of Output Indicators

In China, the efficiency of delivery is low [61]. *Quantity of MSW Collected and Transported* is the amount of MSW that can be transported to waste transfer or treatment stations, which is affected by the collection rate and the transportation rate. It was selected as the first output indicator, which correspond to the first input indicator to measure the efficiency of MSW collection and transportation [38]. We not only considered the scientific indicator selection, but also considered harmlessness in the treatment of MSW in China. We took *MSW Harmless Treatment Rate* as the second output indicator to evaluate MSWM efficiency, and it objectively reflected the level of MSW treatment.

#### 2.3.3. Selection of Environmental Indicators

Environmental indictor is the factor, which affects the efficiency of MSWM but is not subjectively controlled by the sample. Considering the characteristics of MSWM, the paper selected *Quantity of Patent Authorization, Total Retail Sales of Consumer Goods* and *Excellent Rate of Urban Air Quality* as the environmental variables. Previous scholars have found that the level of science and technology have a positive impact on the efficiency of MSWM, and *Quantity of Patent Authorization* better represents the scientific and technological level of city [62]. *Total Retail Sales of Consumer Goods* can measure the consumption level of urban residents, and the increase of consumption ability promotes the increase of MSW generation, which in turn affects the MSWM efficiency [63,64]. *Excellent Rate of Urban Air Quality* is closely related to the environmental condition [65]. With the fewer excellent days of urban air quality and the worse the environmental condition, it is more likely to arouse residents’ self-reflection and improve residents’ environmental protection awareness. Eventually, people are more likely to consciously classify MSW [66].

### 2.4. Sample Selection and Data Collection

In this paper, we selected 34 cities as DMUs. Pilot cities, municipalities and provincial capital cities were included. The eight pilot cities for MSW classification, namely, Beijing, Shanghai, Guangzhou, Shenzhen, Nanjing, Hangzhou, Xiamen and Guilin, were identified by MOHURD in June 2000. The reason why we chose municipalities and provincial capitals was because they had typical significance in economic, social and ecological development. We didn’t analyze Lhasa due to the lack of data on the *Total Retail Sales of Consumer Goods*. Eventually, 33 cities were chosen. To fully understand the status of MSWM in China, we analyzed the MSWM efficiency of 33 typical cities. At the same time, eight pilot cities were compared with other 25 cities to evaluate the pilot effect of MSW classification. From Table 2, we can see the general situation of 33 cities.

*Number of Vehicles and Equipment Designated for Municipal Environmental Sanitation, Fixed Assets Investment in the Public Facilities of Urban Environmental Sanitation, Quantity of MSW Collected and Transported* and *MSW Harmless Treatment Rate* were all from *China Urban Construction Statistical Yearbook*. Not only *Quantity* of *Patent Authorization* but also *Excellent Rate of Urban Air Quality* came from the corresponding urban statistical yearbooks. *Total Retail Sales of Social Consumer Goods* was from the statistics database of *China Economic and Trade Network*. The descriptive summaries of inputs and outputs are shown in Table 3.

### 2.5. Analysis Tools

In the research, DEAP 2.1 (University of New England, New South Wales, Australia), Frontier 4.1 (University of New England, New South Wales, Australia), MATLAB R2017b (MathWorks, Natick, MA, USA) were employed (Table 4).

DEAP 2.1 is the computer program designed for DEA methods: CCR (a DEA model with variable returns to scale, which is proposed by Cooper, Charnes and Rhodes), BCC, the extension of CCR and BCC models, Malmquist [67]. The program is written by Tim Coelli for IBM compatible personal computers [68]. DEAP 2.1 is an upgraded version of DEAP 2.0. It also does not need to be installed. It can be easily run from WINDOWS using file manager. Compared with MAXDEA, DEA-SOLVER, DEAP 2.1 is simpler and more convenient. Hence, it has been widely used in various fields [40,69]. DEAP 2.1 can measure TE, PTE and SE, which satisfies the analysis of this research. In this research, the input and output data of 33 typical cities were analyzed by DEAP 2.1.

Frontier 4.1 is a computer program written by Tim Coelli [70]. It is an upgraded version of Frontier 2.0 and it does not need to be installed. There are many differences between Frontier 4.1 and Frontier 2.0. For example, not only Battese and Coelli (1995) model but also cost functions cannot be estimated in Frontier 2.0. However, in Frontier 4.1, they can be estimated. Frontier 4.1 can provide maximum likelihood estimates of a wide variety of stochastic frontier production and cost functions [71]. It is introduced to eliminate environmental variables and random errors.

MATLAB (matrix laboratory) has become a strong functional mathematic software tool for numerical analysis, engineering and scientific drawing, digital image processing, and so on. And it is widely used in technical computing [72]. It needs to be installed. The FCM algorithm is essentially a continuous iteration of the membership matrix until the termination condition is satisfied [60]. MATLAB is a powerful software for matrix operation [73]. Therefore, MATLAB is used to implement FCM algorithm.

In 1984, MathWorks officially released MATLAB 1.0 (the original version of MATLAB) (MathWorks, Natick, MA, USA). Subsequently, the version of MATLAB is continually upgraded. In September 2017, MathWorks released MATLAB R2017b. MATLAB R2017b is an upgraded version of MATLAB R2017a. It adds important deep learning capabilities that simplify how engineers, researchers, and other domain experts design, train, and deploy models. With the MATLAB R2017b employed, FCM algorithm was applied to cluster analysis of MSWM efficiency.

## 3. Results

**Figure 2:**
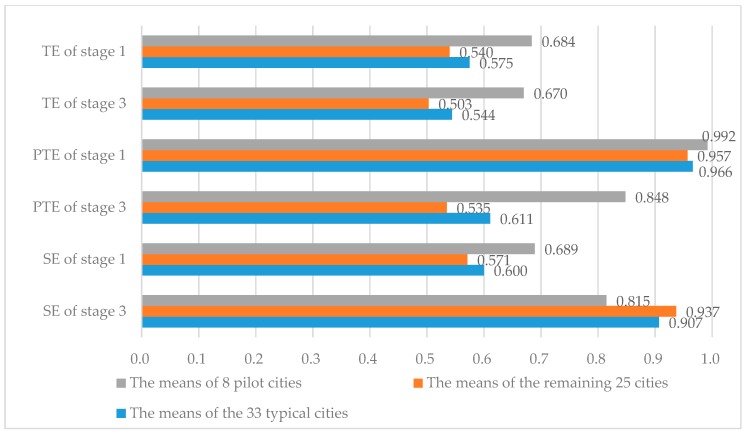
Different types of cities before and after the Adjustment of MSWM efficiency means.

With DEAP2.1 and FRONTIER 4.1 employed, taking 33 typical cities as samples, we developed three-stage DEA model to measure the TE, PTE and SE of MSWM. The results of the efficiency analysis were available in Table 5 and Figure 2.

### 3.1. Stage 1: Traditional DEA

From Table 5 and Figure 2, we achieved two valid results. Without eliminating the influence of environmental factors and random errors, the mean of TE, PTE and SE in eight pilot cities for MSW classified collection was 0.684, 0.992, 0.689, respectively. The average of TE, PTE and SE in 33 typical cities was 0.575, 0.966, and 0.600. The value of TE is not 1 indicates that DMU is non-DEA efficiency. In the preliminary view, the three efficiency mean values of eight cities were higher than the other 25 cities. Therefore, some achievements were obtained in the pilot. Shenzhen, Hangzhou and Guilin were at the frontier of production. Nevertheless, as the “Senior Members” of the pilot cities for MSW classified collection, the TE and SE of Beijing and Shanghai were below the average in China. In order to improve MSWM efficiency, taking action from all aspects is needed. It is necessary to improve management skills, boost technologies and optimize scale.

Among the 33 representative cities, Nanning had the lowest TE. The PTE of other cities was higher than the SE of themselves except Lanzhou. The PTE of the other 31 cities was greater than 0.9, apart from Harbin and Lanzhou. Besides, the SE of 25 cities was less than 0.85. It all signaled that the low SE was the main reason for restricting the MSWM efficiency in China. The value of PTE was not 1, which indicated cities cannot effectively integrate and use inputs [17]. The TE of Shenzhen, Hangzhou, Guilin, Hohhot, Fuzhou, Xining and Yinchuan was 1, the return to scale was constant and reaches the optimal scale. Moreover, the remaining 26 cities were in a state of decreasing returns to scale, and there was room for improvement in terms of PTE and SE.

### 3.2. Stage 2: SFA Regression

For the purpose of analyzing the influence of three environmental variables on the slacks of two input variables, the slacks of Stage 1 were the dependent variables. Three environmental variables were explanatory variables, namely, *Quantity of Patent Authorization*, *Total Retail Sales of Consumer Goods* and *Excellent Rate of Urban Air Quality*. The results of SFA regression were obtained by Frontier4.1, as shown in Table 6.

As we can see from Table 6, the coefficients of the three environmental variables for the slacks of two input variables passed the significance test, which indicated that the environmental variables had a significant impact on the input slack variables. The selection of the model variables appears to be reasonable. LR passed the significance test at 1%, indicating that the model was reasonable and suitable for regression analysis using SFA [50]. The *γ* values of the two input slack variables were both 0.999, which indicated that the influence of management factor was dominant. The *γ* value passed the significance test at 1%. So, the impact of environmental factors and random errors on efficiency was significant. It is necessary to strip out and analyze the environmental factors and random errors by employing SFA model. The results of the impact of environmental factors were as follows:

The correlation between **Z_1_** and the slacks of two input variables were significantly negative, indicating that an increase in scientific and technological level will reduce the input and improve the efficiency of MSWM. The regression coefficient between the slacks of two input variables and household consumption, the slacks of two input variables and environmental condition were both positive and passed the 1% significant test, which confirmed the previous analysis.

According to the analysis of SFA, the MSWM efficiency was affected by external environmental factors and random errors, and the efficiency of Stage 1 was not real efficiency. Consequently, to obtain true and reliable management efficiency, the influence of the environmental variables and random errors should be removed, and 33 typical cities should be placed under the same objective conditions.

### 3.3. Stage 3: Adjusted DEA

Based on the data obtained in the Stage 2, the raw input data was adjusted by using Formula (3). The DEA model was adopted again to analyze the MSWM efficiency. And then, we obtained the adjusted TE, PTE and SE.

It can be seen from Table 5 and Figure 2 that the efficiency values for MSWM in the first and the third stages showed major differences after the elimination of environmental and random factors. The average value TE of the eight pilot cities declined to 0.670 from 0.684, the mean PTE decreased from 0.992 to 0.848. The mean SE increased, rising from 0.689 to 0.815. While the average value TE of 33 typical cities decreased from 0.575 to 0.544, the average PTE decreased from 0.966 to 0.611, and the average SE increased significantly from 0.600 to 0.907. Hence, it is necessary to adjust the raw input data. The actual level of MSWM was overvalued. Whether for eight pilot cities or the remaining 25 cities, the main restriction on the efficiency of MSWM was PTE.

In terms of the TE, there was little change in 33 typical cities. Among eight pilot cities for the classified collection of MSW, only Hangzhou and Guilin had a TE of 1. Shenzhen’s TE decreased from 1 to 0.917. The TE of Beijing and Shanghai was still at a low level. The mean TE of the eight pilot cities for the classified collection of MSW was higher than the mean of the other 25 cities. From a national perspective, the TE of six cities has increased, and the average growth rate was more than 10%. Among the six cities, Haikou had the largest growth rate of 59.52%. It showed that the poor environment of MSW was one of the reasons for the low efficiency of Stage 1 in Haikou [49]. After the adjustment, the number of cities with TE reaching the effective value was reduced from 7 to 5. Hangzhou, Guilin, Hohhot, Fuzhou and Xining were still at the production frontier, while Shenzhen and Yinchuan were no longer at the production frontier, which indicates that Shenzhen and Yinchuan have excellent and suitable environmental factors for MSWM. Therefore, Hangzhou, Guilin, Hohhot, Fuzhou and Xining were the benchmark of cities with the efficiency score of less than 1 [58]. The TE of 17 cities, including Beijing and Shenzhen, with an average decline of 6.51%, indicating that these cities were “cared for” by good environmental factors and the actual management level was overvalued [52]. The decreasing rate of Haikou was the largest, which was up to 37.35%. The overall TE of MSWM in China was not high, with an average of 0.544.

As for PTE, in eight pilot cities, the PTE of Guangzhou, Nanjing and Xiamen decreased by 39.21% on average. The reduction of PTE indicated that the efficiency of using input elements was lower than it seemed. In Stage 1, the higher PTE was closely related to the favorable environment. And the remaining five cities was 1. That is, 5 cities can fully integrate and use input factors [56]. What is more, the mean PTE of the eight pilot cities was higher than the other 25 cities. Therefore, from the perspective of PTE, the results achieved by the pilot cities was worthy of affirming. Among the 33 typical cities, only Lanzhou was increased by 0.05 after its adjustment. Before and after adjustment, the PTE of eight cities was 1, while the other 24 cities declined in PTE. The largest decline was 87.07% in Nanning, and the mean descent rate of 24 cities was 47.04%. It demonstrated that PTE of the 24 cities was overestimated. Ten cities fell from the production frontier of technical before the adjustment to the non-production frontier, including Beijing, Nanchang, etc.

With regard to the SE, the mean SE of the eight pilot cities for the classified collection of MSW was 0.815, which was smaller than 0.937 (the average of the other 25 cities). Obviously, the SE of eight pilot cities needed to be improved. Among eight pilot cities, there was a gap between the actual scale and the optimal production scale of the remaining six pilot cities. The 6 cities must explore the optimal scale except Hangzhou and Guilin [57]. According to the analysis of the national 33 typical cities, the SE of Hangzhou, Guilin, Hohhot, Fuzhou and Xining remained unchanged at 1. The SE of Beijing, Shenzhen, Shijiazhuang, Lanzhou and Yinchuan were reduced by 1.18%, 8.3%, 0.85%, 21.48% and 2.1%, respectively. The SE of these cities benefited from good environment [57]. The SE of other 23 cities was increased by varying degrees, with an average growth of 145.80%. Nanning’s growth rate was as high as 673.32%, followed by Wuhan, Urumqi and Tianjin, which were 459.89%, 352.05% and 278.24%.

In the aspect of the changes in returns to scale, among the eight pilot cities, Hangzhou and Guilin were in a state of constant returns to scale. Xiamen was increasing returns to scale. While the remaining five cities were at the decreasing stage of the scale return, the scale of input can be appropriately expanded. The returns to scale of the 33 representative cities before and after adjustment were quite different. In the Stage 1 of the traditional DEA model, seven cities were in a state of constant returns to scale, accounting for up to 21.21% of the total, and the remaining 26 cities were decreasing returns to scale. After the adjustment, 18 cities were decreasing returns to scale, the proportion decreased from 78.79% to 54.54%. Six cities were in a state of constant returns to scale. And the remaining nine cities were at the increasing returns to scale. It can be explained that the main restriction on the efficiency of MSWM was not the scale reduction [74].

### 3.4. Cluster Analysis of MSWM Efficiency

**Figure 3:**
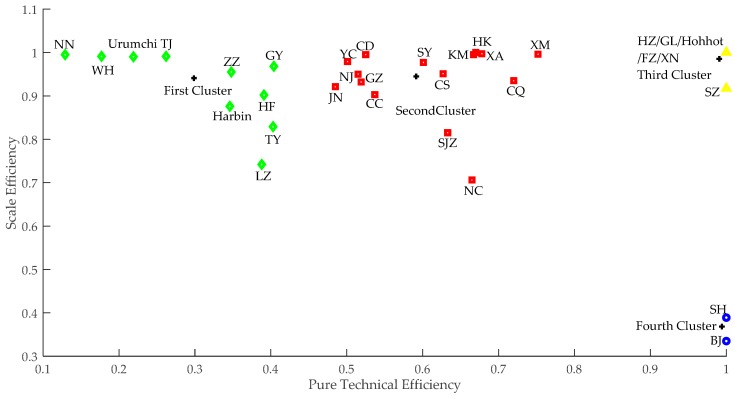
Clustering Diagram of MSWM.

To better distinguish the efficiency of MSWM in each city, cluster analysis was conducted on 33 typical cities using the data obtained in Stage 3. With the MATLAB R2017b employed, FCM algorithm was applied to cluster analysis of MSWM efficiency. The clustering diagram was shown in Figure 3.

According to the clustering results, the efficiency of MSWM in the sample cities can be divided into four types. Ten cities, including Nanning, Wuhan, Urumqi, Tianjin, Zhengzhou, Harbin, Guiyang, Hefei, Taiyuan and Lanzhou were the first type named “low-high”. The central coordinate of the first cluster was (0.299, 0.941). Jinan, Yinchuan, Nanjing, Chengdu, Guangzhou, Changchun, Shenyang, Changsha, Shijiazhuang, Nanchang, Kunming, Haikou, Xi’an, Chongqing and Xiamen were the second type named “medium-high”. The cluster center coordinate was (0.592, 0.945). Hangzhou, Guilin, Hohhot, Fuzhou, Xining and Shenzhen belonged to the third type named “high-high”, and the cluster center coordinate was (0.991, 0.985). While Beijing and Shanghai were the fourth type named “high-low”, the cluster center coordinate was (0.994, 0.368). Hence, among the eight pilot cities, Nanjing, Guangzhou and Xiamen belonged to the “medium-high” type. Hangzhou, Guilin and Shenzhen were the “high-high” type, while Beijing and Shanghai were the “high-low” type.

## 4. Discussion

In 1990s, the concept of MSW classification came into China. Since then, Chinese government has always emphasized MSWM to avoid economic and environmental problems caused by MSW. Identifying the status of MSWM, finding high-efficiency MSWM policy implementation routes and the breakthrough on improving MSWM efficiency were China’s important tasks [29]. The results of Stage 2 showed that the level of science and technology, the consumption level of urban residents, and environmental condition have impacts on the efficiency of MSWM. The results confirmed the expectations. Among them, the higher level of science and technology was conducive to saving investment [75]. The higher the level of science and technology, the more likely to use Internet Technology to innovative intelligent delivery mode, intelligent collection and transportation mode. In addition, overcoming the technical difficulties of MSW classification and treatment was beneficial to improve the efficiency of MSWM [56]. Urban residents’ consumption level and environmental condition were positive to two slacks of input variables. The results indicated that higher urban residents’ consumption level and environmental condition were not conducive to improving management efficiency [76]. The higher consumption level of urban residents, the greater *Total Retail Sales of Social Consumer Goods* [77]. Then, the more MSW generated, the more unfavorable to the improvement the efficiency of MSWM [77]. The worse environment condition was, the less likely it was to waste material and financial resources. We tentatively put forward poor environmental conditions aroused residents’ awareness of environmental protection, which was beneficial for residents to start from themselves, actively participated in the whole process of MSW classification. That, in turn, can save material and financial resources, and bring positive impact on MSWM efficiency.

After excluding the environmental factors and random errors, the TE average value of 33 typical cities declines to 0.544 from 0.575. The efficiency of MSWM in China was not high, and the effectiveness of MSW classification was not obvious. The mean PTE value in Stage 3 is 0.611. The low efficiency of MSWM is constrained by low PTE. The main reasons may be that the implementation of MSW classification was insufficient, the level of MSW disposal technology was not high, and the quality and quantity of classification facilities in China were insufficient. China should attach importance to improving technological level and managerial skills for the purpose of improving the efficiency of MSWM.

At present, the eight pilot cities for classified collection of MSW, including Beijing and Shanghai and so on, have been trialed for 18 years. But the results showed that the effectiveness was not significant. Only Hangzhou and Guilin were at production frontier, indicating only two of the eight pilot cities took the leading position in China in terms of MSWM. The average value TE of the eight pilot cities was bigger than the remaining 25 cities. Moreover, the mean of SE was smaller than the remaining 25 cities. The results indicated that the TE of the eight pilot cities was subject to the lower SE. Management skills and technical level of 25 cities need to be improved.

Comparing the results of the Stage 1 with the Stage 3, Haikou had the largest growth in TE, which indicated that Haikou was in bad environment. Haikou municipal government should increase the investment in MSWM and increase the publicity of MSW classification. It is necessary to improve the classification awareness of residents and further create a good atmosphere for MSWM. PTE was declined in 24 cities, indicating that the surface PTE was higher than the real PTE. And the PTE of MSWM in 24 cities benefited from the environment. In terms of SE, the SE of other 23 cities was increased. The economies of scale of 23 cities were adversely affected by environment. Therefore, it is necessary to introduce three-stage DEA model.

**Figure 4:**
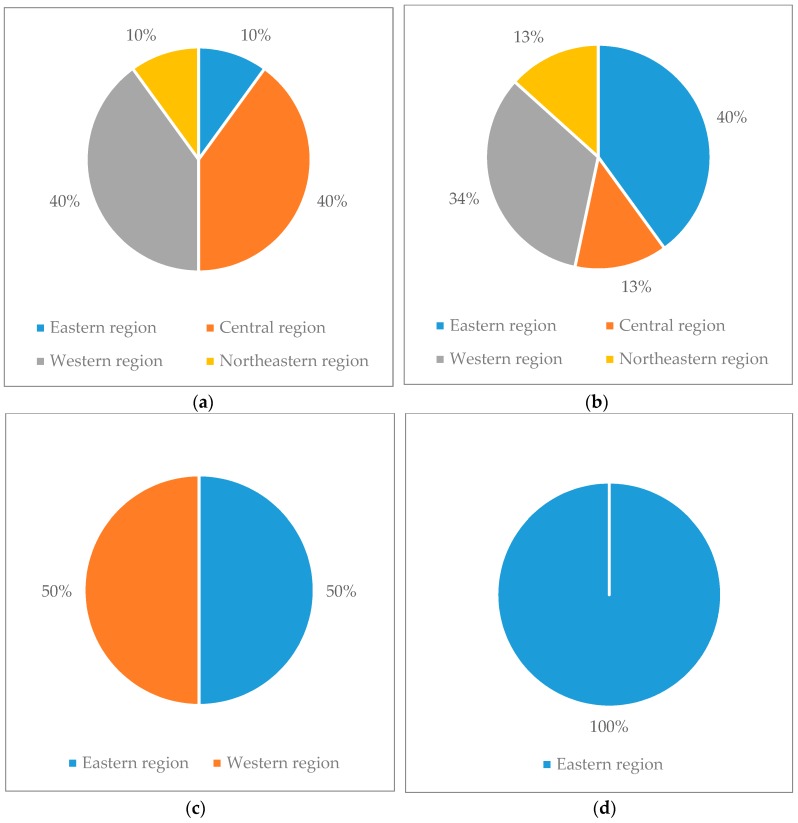
Regional distributions of four types of MSWM efficiency. (**a**) Regional distribution of the first type of MSWM efficiency; (**b**) Regional distribution of the second type of MSWM efficiency; (**c**) Regional distribution of the third type of MSWM efficiency; (**d**) Regional distribution of the fourth type of MSWM efficiency.

Regional distribution of four types of MSWM efficiency is available in Figure 4.

As we can see from Figure 4, from the perspective of categories, the first type of cities were the cities mostly in the western and central of China, with a total ratio of 80%. And the PTE of these cities needed to be improved. In the second type, there were many cities in the eastern and western of China, with a total ratio of 74%. The third type of cities had the highest PTE and SE. The eastern region cities like Shenzhen, Hangzhou, Fuzhou and the western region cities like Guilin, Xining and Hohhot belonged to the third type. The fourth type of cities was all eastern cities, and the TE was constrained by scale constraints. These cities were more advanced in terms of technology, organization and the methods of management. Therefore, the distribution of MSWM efficiency was not balanced. From the perspective of regional scope, the cities in the northeast were distributed in the first and second types. The SE was rather high, and the PTE needed to be improved. The distribution in the eastern region was more scattered and distributed in four types. 2/3 of the central cities belonged to the first type and the remaining belonged to the second type and all the central cities’ SE were high. In the western cities, other cities belonged to the first and second type, except Guilin, Xining and Hohhot. And the PTE of these cities were not high. The PTE of the central and northeastern regions was relatively low. The SE of the western region was relatively high and the efficiency distribution of the eastern region was relatively scattered. We can draw a conclusion that there were regional differences in the efficiency of MSWM.

### 4.1. Countermeasures to Improve the Efficiency of MSWM

The efficiency value calculated in Stage 3 was more realistic. So, the countermeasures to improve the efficiency of MSWM in China were put forward based on the efficiency value of Stage 3 [58]. It is significant that finding the breakthrough on improving MSWM efficiency. For the four types of cities, differential management should be implemented according to the actual situation of each city.

As for the first “low-high” type and the second “medium-high” type of cities with low PTE, Improving PTE is a top priority. Not only the scientific and technological level of MSW classification but also the management skills and decision-making level should be improved.

As for the first and second type of cities, improving the scientific and technological level is the first key solution. Improving the technical level of the relevant staff [78] and strengthening scientific research, tackling key research projects are essential [79]. They should make full use of Internet Technology and combine reality with Exploitation and Innovation. In terms of classified delivery, they should explore new patterns such as “community worker and volunteers”, “professional enterprises, professionals and intelligent devices”, etc. In the case of classified collection, MSW bins which are suitable for household and office MSW sorting should be developed. As for classified transportation, they should establish an intelligent collection and transportation network which is linked with classified delivery. These cities also need to develop MSW sorting recycling platform which can integrate online and offline [80]. In terms of classified disposal, they should make efforts to tackle different MSW disposal technologies and remold existing MSW disposal facilities [81].

In terms of the first and second types of cities, advocating multi-subject governance is the second key solution. All subjects are encouraged to make joint efforts. The MSWM is a common career of the whole society. According to China actual situation, MSWM should be led by the government [82]. Specifically, the government should formulate relevant policies and regulations so that it provides a pathway for multi-subject to participate in MSWM [61]. In addition, in order to encourage social resource to participate in MSWM, actively exploring good ways such as franchise and operating lease is required. As for the government, it is also a good measure to explore innovative models. Enterprises and social organizations are encouraged to carry out MSWM services. Gradually introducing the main subject of MSW classification into the environmental credit system is also needed. Furthermore, the government should guide the industrialization of MSW disposal and incorporate it into regional development planning [63]. Moreover, the government can’t ignore the propaganda. Government can adopt the propaganda model of “education and guidance” to strengthen the awareness of the people’s MSW classification. It is necessary to enhance residents’ enthusiasm for participating in the supervision of enterprises and government behavior. At the same time, the government must attach great importance to coordinate the interests of all parties.

As for the fourth “high-low” type of cities with low SE of MSWM, paying attention to narrowing the gap between actual scale and optimal scale is required [56]. Beijing and Shanghai were at the stage of decreasing returns to scale. The two cities should optimally allocate resources and focus on sustainable development [83]. At the same time, ensuring the efficiency of input elements is also important.

In addition to ensuring leading position, the third type of “high-high” cities with high PTE and TE should keep pace with the times. Moreover, it is essential that improving the efficiency of the pilot cities and encouraging the eight pilot cities to play a demonstration role in MSWM. For the pilot cities of MSW classification, Hangzhou, Guilin and Shenzhen should constantly introduce new management skills and technology. The governments of these cities should innovate the propaganda model of MSW classification to strengthen the awareness of the people’s MSW classification. Management subjects should actively learn from the international advanced management skills. And constantly explore the model of enterprises participating in MSWM is essential, such as BT (Build-Transfer), ROT (Renovate-Operate-Transfer), BOO (Build-Own-Operate). As for Beijing and Shanghai, it is necessary to enhance the SE by optimizing allocation of resources. Guangzhou, Nanjing and Xiamen should pay great attention to promote the PTE with new technology and higher management capabilities. Paying attention to explore and give full play to the leading role of the eight pilot cities in MSW classification is of great significance for China to improve the MSWM efficiency.

For cities with decreasing returns to scale, the allocation of resources should be optimized. The cities have two important things which are increasing the investment in technology and adjusting the scale of investment [84]. In order to improve utilization efficiency, it is urgent that optimizing the combination of production factors according to market needs. Cities with increasing returns to scale should take the path of expanding investment scale. These cities should follow national policies and implement mandatory classification of MSW to expand the scope of MSW classification.

### 4.2. Strengths and Limitations

This paper innovatively introduced the three-stage DEA model into the research, and the MSWM efficiency of China typical cities in 2016 was accurately evaluated. The paper is helpful for decision-makers to identify the status of MSWM and put forward effective countermeasures. Furthermore, it is beneficial to improve public health.

This research method can be adopted to measure the efficiency of MSWM in many years so that the MSWM efficiency of China in long period can be reflected more accurately. And the model can be used to evaluate MSWM efficiency of cities in other countries. This research has potential for further developments. Firstly, owing to the limitations of data acquisition, the research on the recycling, harmlessness and reduction effects of MSW classification in China cannot be carried out. Therefore, our next step is to dig deeper into the currently available data so that we can find the best substitution indicators. Secondly, our research is unable to cover all considerations due to the unavailability of data. We will quantify the social factors in the concept of sustainable development and incorporate them into the evaluation system of MSWM. Thirdly, we can analyze the efficiency of MSWM from the smaller perspective, such as the status of MSWM in enterprises and social organizations.

## 5. Conclusions

Poor public health is always associated with the mismanagement of MSW. In China, more importance has been attached to MSWM to improve public health. However, there are still some problems in every link of MSWM. The research shows that China’s MSWM efficiency is low. The effectiveness of policy implementation is expected to be improved. The management effectiveness of eight pilot cities (MSW classification) is affirmative but not that significant. There are regional differences in the efficiency of MSWM in China. The PTE of the central and northeastern regions is relatively low, while the SE of the western region is higher than other regions. In addition, the efficiency distribution of the eastern region is scattered.

In order to improve the efficiency of MSWM, differential management should be implemented according to the actual situation of each city. Different types of cities have different breakthroughs on improving MSWM efficiency. As for the first “low-high” type and the second “medium-high” type of cities with low PTE, the breakthrough to improve efficiency is improving PTE. In the process of policy implementation, these cities should pay attention to improving the technical level of the relevant staff, introducing new technology for MSW classification, advocating multi-subject governance. Because the fourth type of “high-low” cities with low SE are at the decreasing stage of the scale return, the key point for high-efficiency MSWM policy implementation routes is narrowing the gap between actual scale and optimal scale. To optimize the allocation of resources, enterprises and social organizations should be encouraged to participate in MSWM. The third “high-high” type of cities is the most ideal. The outputs are larger than other types with given inputs. New methods and management skills should also be constantly explored. Innovating the propaganda model of MSW classification is vital. It is feasible to enhance the publicity effect by developing on-the-spot presentation, thematic activities, and so on. Management subjects should actively learn from the international advanced management skills. Enterprises are supposed to actively explore the model of participation in MSWM, such as BT, ROT, BOO. Moreover, we should also keep exploring and playing the role of “leading-sheep” in the pilot cities.

## Figures and Tables

**Table 1:** Indicator system for measuring the efficiency of municipal solid waste (MSW) classification.

Indicators	Variables	Unit
Input indicators (X)	Number of Vehicles and Equipment Designated for Municipal Environmental Sanitation (X_1_)	Number
	Fixed Assets Investment in the Public Facilities of Municipal Environmental Sanitation (X_2_)	10,000 RMB
Output indicators (Y)	Quantity of MSW Collected and Transported (Y_1_)	10,000 ton
	MSW Harmless Treatment Rate (Y_2_)	Percentage
Environmental indicators (Z)	Quantity of Patent Authorization (Z_1_)	Piece
	Total Retail Sales of Social Consumer Goods (Z_2_)	100,000,000 RMB
	Excellent Rate of Urban Air Quality (Z_3_)	Percentage

**Table 2:** Overview of 33 typical cities in 2016.

City	UDRP ^1^	X_1_	X_2_	NHTP/G ^2^	HTP ^3^	Y_1_	QHT ^4^	Y_2_
Beijing *^,5^	2172.90	11,033	1,613,451	27	24,341	872.61	871.20	99.84
(BJ)								
Shanghai *	2419.70	7036	65,828	14	23,530	629.37	629.37	100
(SH)								
Guangzhou *	1759.49	4550	141,734	6	12,727	504.36	484.67	96.10
(GZ)								
Shenzhen *	1190.84	2456	102	8	14,025	572.28	572.28	100
(SZ)								
Nanjing *	703.05	1925	112,355	7	8950	212.72	212.72	100
(NJ)								
Hangzhou *	899.96	1484	350	6	6007	342.46	342.46	100
(HZ)								
Xiamen *	520.08	1028	50,002	5	3760	166.21	162.48	97.75
(XM)								
Guilin *	139.15	455	2496	1	1000	40.86	40.86	100
(GL)								
Tianjin	1360.43	4449	18,518	9	10,800	269.03	253.30	94.16
(TJ)								
Shijiazhuang	472.74	962	1937	6	4100	95.97	95.97	100
(SJZ)								
Taiyuan	387.00	2837	3859	2	4727	180.98	180.98	100
(TY)								
Hohhot	194.53	444	5784	2	1550	60.38	60.38	100
Shenyang	649.73	1931	30,771	3	5135	263.03	262.90	99.95
(SY)								
Changchun	479.19	4063	180	5	5593	194.89	175.93	90.27
(CC)								
Harbin	620.17	3118	6973	4	3380	163.00	142.23	87.26
Hefei	450.55	1920	27,943	2	4527	144.30	144.30	100
(HF)								
Fuzhou	275.41	608	4027	2	2850	108.24	107.16	99
(FZ)								
Nanchang	336.93	1261	6000	1	2380	96.07	96.06	99.99
(NC)								
Jinan	481.52	1651	18,303	3	3168	167.32	167.32	100
(JN)								
Zhengzhou	748.27	2846	15,015	2	4700	223.08	223.08	100
(ZZ)								
Wuhan	1121.62	8748	122,619	8	9650	356.29	356.29	100
(WH)								
Changsha	351.51	1592	29,030	1	7111	215.28	215.28	100
(CS)								
Nanning	539.78	4748	56,383	3	3400	107.31	106.28	99.04
(NN)								
Haikou	262.60	3603	745	2	3400	94.51	94.51	100
(HK)								
Chongqing	2907.38	3116	55,194	24	11,753	494.13	494.05	99.98
(CQ)								
Chengdu	940.54	2687	736	4	7800	350.96	350.96	100
(CD)								
Guiyang	319.00	1775	9182	2	3000	118.74	113.99	96
(GY)								
Kunming	443.41	1429	5113	6	5380	200.92	194.86	96.98
(KM)								
Xi’an	629.24	1916	20,771	4	9683	346.81	345.77	99.7
(XN)								
Lanzhou	268.85	1330	44,053	4	3114	96.08	32.43	33.75
(LZ)								
Xining	147.02	352	19,170	3	1360	56.16	53.56	95.36
(XN)								
Yinchuan	151.81	1033	400	2	2500	46.78	45.38	97
(YC)								
Urumchi	312.34	3279	39,118	2	3636	145.06	138.81	95.69

Note: ^1^ UDRP: Urban District Resident Population (10,000 persons); ^2^ NHTP/G: Number of Harmless Treatment Plants/Grounds; ^3^ HTP: Harmless Treatment Capacity (ton/day); ^4^ QHT: Quantity of Harmlessly Treated (10,000 ton); ^5^ Cities with “*” are pilot cities for MSW classification.

**Table 3:** Descriptive summaries of inputs and outputs in 2016.

Variable	Mean	St. Dev	Min	Max
X_1_	2778	2371.74	352	11,033
X_2_	76,610.36	278,296.35	102	1,613,451
Y_1_	240.49	190.83	40.86	872.61
Y_2_	96.30	11.62	33.75	100
Z_1_	20,482.48	23,911.34	829	100,578
Z_2_	3343.56	2784.08	346.85	11,005.10
Z_3_	74.36	16.27	43.44	99.40

**Table 4:** Analysis tools used in the research.

Analysis Tool	Inventor	Main Characteristics	Role in the Research
DEAP 2.1	Tim Coelli	DEAP 2.1 is the computer program designed for DEA methods: CCR, BCC, the extension of CCR and BCC models, Malmquist. It does not need to be installed. Compared with MAXDEA, DEA-SOLVER, it is simpler and more convenient.	Analyzing the TE, PTE and SE of MSWM
Frontier 4.1	Tim Coelli	Frontier 4.1 is a computer program. It does not need to be installed. It can provide maximum likelihood estimates of a wide variety of stochastic frontier production and cost functions.	Eliminating the influence of environmental factors and random errors
MATLAB R2017b	MathWorks	MATLAB R2017b is a strong functional software for matrix operation. It needs to be installed. It adds new important deep learning capabilities that simplify how engineers, researchers, and other domain experts design, train, and deploy models.	Clustering analysis of 33 typical cities

**Table 5:** The 33 typical cities in China before and after adjustment of municipal solid waste management (MSWM) efficiency.

City	Stage 1				Stage 3			
	TE	PTE	SE	RTE	TE	PTE	SE	RTE
Beijing *^,^^1^	0.339	1	0.339	drs	0.335	1	0.335	drs ^2^
(BJ) ^3^								
Shanghai * (SH)	0.384	1	0.384	drs	0.389	1	0.389	drs
Guangzhou *	0.476	0.961	0.496	drs	0.483	0.519	0.932	drs
(GZ)								
Shenzhen * (SZ)	1	1	1	-	0.917	1	0.917	drs
Nanjing *	0.506	1	0.506	drs	0.489	0.515	0.95	drs
(NJ)								
Hangzhou *	1	1	1	-	1	1	1	- ^4^
(HZ)								
Xiamen *	0.766	0.977	0.784	drs	0.749	0.752	0.996	irs ^5^
(XM)								
Guilin *	1	1	1	-	1	1	1	-
(GL)								
Tianjin	0.267	0.942	0.283	drs	0.26	0.262	0.991	irs
(TJ)								
Shijiazhuang	0.822	1	0.822	drs	0.515	0.633	0.815	drs
(SJZ)								
Taiyuan	0.346	1	0.346	drs	0.334	0.403	0.829	drs
(TY)								
Hohhot	1	1	1	-	1	1	1	-
Shenyang	0.607	0.999	0.607	drs	0.587	0.601	0.977	drs
(SY)								
Changchun	0.54	0.903	0.598	drs	0.485	0.537	0.903	irs
(CC)								
Harbin	0.26	0.873	0.298	drs	0.303	0.346	0.876	irs
Hefei	0.374	1	0.374	drs	0.353	0.391	0.902	drs
(HF)								
Fuzhou	1	1	1	-	1	1	1	-
(FZ)								
Nanchang	0.487	1	0.487	drs	0.47	0.665	0.706	drs
(NC)								
Jinan	0.49	1	0.49	drs	0.447	0.485	0.921	drs
(JN)								
Zhengzhou	0.361	1	0.361	drs	0.333	0.348	0.955	drs
(ZZ)								
Wuhan	0.176	1	0.176	drs	0.175	0.177	0.991	drs
(WH)								
Changsha	0.618	1	0.618	drs	0.596	0.627	0.951	drs
(CS)								
Nanning	0.125	0.99	0.126	drs	0.128	0.129	0.995	drs
(NN)								
Haikou	0.42	1	0.42	drs	0.67	0.67	1	-
(HK)								
Chongqing	0.682	1	0.682	drs	0.673	0.72	0.935	drs
(CQ)								
Chengdu	0.566	1	0.566	drs	0.522	0.525	0.995	drs
(CD)								
Guiyang	0.367	0.96	0.383	drs	0.391	0.404	0.968	irs
(GY)								
Kunming	0.662	0.97	0.683	drs	0.663	0.667	0.995	irs
(KM)								
Xi’an	0.784	0.997	0.786	drs	0.676	0.678	0.997	drs
(XN)								
Lanzhou	0.319	0.338	0.945	drs	0.288	0.388	0.742	irs
(LZ)								
Xining	1	1	1	-	1	1	1	-
(XN)								
Yinchuan	1	1	1	-	0.49	0.501	0.979	irs
(YC)								
Urumchi	0.219	0.957	0.229	drs	0.217	0.219	0.99	irs
Mean	0.575	0.966	0.600		0.544	0.611	0.907	

Note: ^1^ Cities with “*” are pilot cities for MSW classification; ^2^ “drs” represents diminishing returns to scale; ^3^ The contents in brackets are the abbreviations for corresponding city names; ^4^ “-” represents constant returns to scale; ^5^ “irs” represents increasing returns to scale.

**Table 6:** Stage 2: Results of Stochastic Frontier Analysis (SFA) regression model.

Variables	Z_1_	Z_2_	Z_3_	Constant	*γ*	LR
Slack Variable of X_1_	−0.006 ***	4.191 ***	0.048 ***	−478.819 ***	0.999 ***	28.848 ***
Slack Variable of X_2_	−0.278 **	255.736 ***	2.670 ***	−30,690.464 ***	0.999 ***	20.944 ***

Note: “***” donates significant at significance levels of 1%; “**” donates significant at significance levels of 5%.
